# Probing phonon dynamics with multidimensional high harmonic carrier-envelope-phase spectroscopy

**DOI:** 10.1073/pnas.2204219119

**Published:** 2022-06-15

**Authors:** Ofer Neufeld, Jin Zhang, Umberto De Giovannini, Hannes Hübener, Angel Rubio

**Affiliations:** ^a^Max Planck Institute for the Structure and Dynamics of Matter and Center for Free-Electron Laser Science, 22761 Hamburg, Germany; ^b^Dipartimento di Fisica e Chimica—Emilio Segrè, Università degli Studi di Palermo, I-90123 Palermo Italy;; ^c^Nano-Bio Spectroscopy Group, Universidad del País Vasco UPV/EHU, 20018 San Sebastián, Spain;; ^d^Center for Computational Quantum Physics (CCQ), The Flatiron Institute, New York, NY 10010

**Keywords:** HHG, ultrafast spectroscopy, phonons, nonlinear optics, pump-robe spectroscopy

## Abstract

We explore pump-probe high harmonic generation (HHG) from monolayer hexagonal-boron-nitride, where a terahertz pump excites coherent optical phonons that are subsequently probed by an intense infrared pulse that drives HHG. We find, through state-of-the-art ab initio calculations, that the structure of the emission spectrum is attenuated by the presence of coherent phonons and no longer comprises discrete harmonic orders, but rather a continuous emission in the plateau region. The HHG yield strongly oscillates as a function of the pump-probe delay, corresponding to ultrafast changes in the lattice such as specific bond compression or stretching dynamics. We further show that in the regime where the excited phonon period and the pulse duration are of the same order of magnitude, the HHG process becomes sensitive to the carrier-envelope phase (CEP) of the driving field, even though the pulse duration is so long that no such sensitivity is observed in the absence of coherent phonons. The degree of CEP sensitivity versus pump-probe delay is shown to be a highly selective measure for instantaneous structural changes in the lattice, providing an approach for ultrafast multidimensional HHG spectroscopy. Remarkably, the obtained temporal resolution for phonon dynamics is ∼1 femtosecond, which is much shorter than the probe pulse duration because of the inherent subcycle contrast mechanism. Our work paves the way toward routes of probing phonons and ultrafast material structural changes with subcycle temporal resolution and provides a mechanism for controlling the HHG spectrum.

## Significance

High harmonic generation (HHG) has recently been established as a powerful method for probing ultrafast electron dynamics in solids. However, it remains unknown if HHG can be similarly applied for probing lattice distortions such as phonons. Specifically, it is unclear if the extreme nonlinearity of HHG can contribute to enhanced temporal resolution or sensitivity for probing lattice dynamics (compared to other, perturbative, methods). Here, we theoretically explore HHG in solids with active phonons. We present a pump-probe and multidimensional spectroscopy approach that relies on carrier-envelope-phase-sensitivity, in which HHG is highly sensitive for phonon dynamics. Strikingly, the predicted temporal resolution is ∼1 femtosecond, much below the probe pulse duration, owing to the subcycle nature of the approach.

High harmonic generation (HHG) has recently been established in condensed matter as a source of coherent extreme ultraviolet radiation (1–3), as well as a useful probe for various material properties such as band structure (4, 5), symmetry (6–8), berry phases (9), topology (10–15), and more. The extremely nonlinear nature of the process can act as a unique platform with enhanced sensitivity for ultrafast dynamics on femtosecond (fs) and sub-fs timescales, which has primarily been applied for exploring driven electron dynamics (16–20). In contrast, HHG spectroscopy as a probe for phonon and lattice dynamics has not been thoroughly studied. It was only recently shown for lower-order third and fifth harmonics that HHG can be sensitive to coherent phonons dynamics (21), though the different physical coupling mechanisms have not yet been identified or analyzed. Here, HHG could ideally be employed as a sensitive probe for ultrafast phonon motion with a plethora of possible physical applications, including lattice-induced symmetry breaking (22), phase transitions (21, 23–26), and energy transfer through nonradiative processes (27).

One common notion is that phononic effects should be quite small in solid HHG because these would average out over many lattice unit-cells, and because phonons typically propagate on picosecond timescales that are much longer than the femtosecond dynamics that standardly drive the HHG process. This notion is also supported by HHG experiments in the gas phase, where molecular vibrations have been shown to only weakly affect HHG (28–30) (causing yield modulations smaller than ∼10%). Nonetheless, driving coherent phonons in a pump-probe setup does not necessarily follow the above rule of thumb since: 1) if the driven dynamics are coherent, their effects would not necessarily average out, and 2) solid phases offer a much larger wealth of phononic band excitations than molecules, where the different underlying HHG mechanisms could lead to enhanced nonlinear responses.

Here, we theoretically investigate optical phonon-assisted HHG in a pump-probe setup. Monolayer hexagonal-boron-nitride (hBN) is coherently pumped with terahertz (THz) light that initiates strong lattice vibrational motion, which is thereafter probed by intense infrared (IR) pulses that generate high harmonics. The electronic and ionic dynamics are described by state-of-the-art ab initio methods and analyzed with respect to the pump-probe delay. In the presence of coherent phonons, the HHG spectrum comprises a continuous emission rather than a well-defined frequency comb. This effect results from the introduction of the long-duration phonon timescale to the system that breaks the time-translational symmetry of the IR pulse and vanishes if phonons are assumed to have random phases (as occurs in the thermal case). We show that the HHG yield strongly, and periodically, oscillates with the pump-probe delay, which directly correlates to the instantaneous structural changes in the lattice such as bond compression or stretching (that are inherently connected to the instantaneous electronic structure). Moreover, the phonon motion results in strong carrier-envelope-phase (CEP) sensitivity in the HHG yield that is absent in the nonpumped system and that standardly does not appear from such multicycle driving (31–35). We demonstrate that the degree of CEP sensitivity versus the pump-probe delay is a very selective measure for femtosecond structural dynamics, which opens routes for HHG spectroscopy with subcycle temporal resolution.

## Results and Discussion

**Fig. 1.**
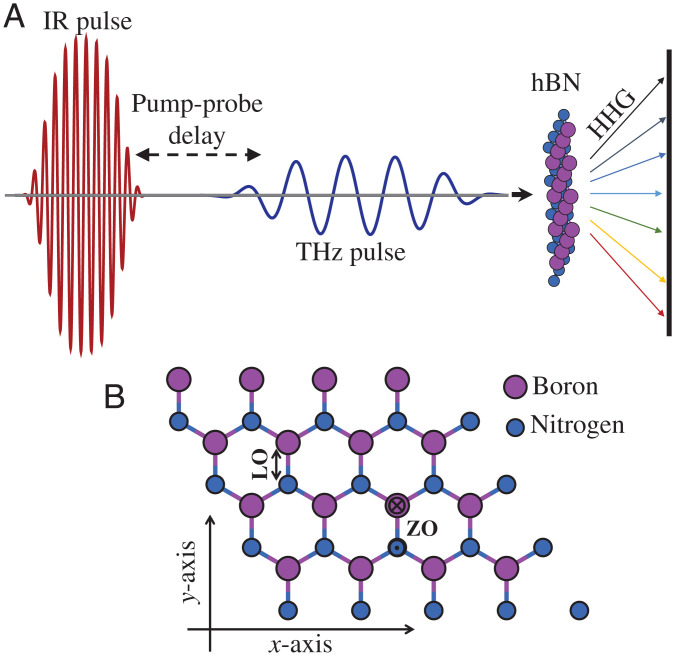
Schematic illustration of the pump-probe HHG setup. (*A*) A THz pulse excites coherent phonon dynamics in hBN, which are subsequently probed by an intense IR pulse that is polarized in the hBN plane and drives HHG. The harmonic yield is measured with respect to the pump-probe delay and optionally also with respect to the IR pulse CEP and polarization. (*B*) Illustration of hBN structure and explored phononic landscape. Ionic vibrations along the *y* axis represent LO modes, while motion out of plane represents ZO modes. The HHG yield is modulated due to changes in the lattice structure. For the LO mode, when the B–N bonds along the *y* axis are compressed, the second set of B–N bonds in the unit cell (that are rotated by 120° from the *y* axis) are stretched, and vice versa.

We begin by describing the simulated setup and theoretical approach. Monolayer hBN is described using density functional theory (DFT) within the Kohn-Sham (KS) formulation and the local-density approximation (LDA), with two in-plane periodic dimensions and an out-of-plane nonperiodic axis. All calculations are performed within the open-access real-space-based octopus code (36–38). Technical details can be found in the *Materials and Methods* section, while specific details about the laser pulse and phonons involved are outlined below. hBN is assumed to interact with a pump THz pulse that excites coherent phonon dynamics, which are subsequently probed by an intense IR laser pulse that generates high harmonics (see Fig. 1 for illustration). The phononic excitation is approximated here by directly initiating the lattice motion rather than describing the interaction with the pump THz pulse, which substantially reduces the computational load. The ionic motion is described by Ehrenfest dynamics (39), where forces are calculated according to the interactions of ions with the laser, the surrounding electron density, and nearby nuclei. The electronic degrees of freedom are described fully quantum mechanically within time-dependent DFT (TDDFT) in the adiabatic approximation (40), in the velocity gauge, and in the dipole approximation. Pseudopotentials are employed to deal with deep core states (41) (see the *Materials and Methods* section for details). Notably, this state-of-the-art framework fully incorporates electron–phonon and phonon–phonon couplings and is thus suitable for studying this complex system.

In this pump-probe setup, there are two main degrees of freedom of interest: the properties of the excited phonon modes and the properties of the HHG driving probe laser pulse. Depending on these, the dynamics and observables can greatly vary. We study here the excitation of in-plane longitudinal optical (LO) phonon modes and out-of-plane optical (ZO) modes, which have slightly different physical properties (see illustration in Fig. 1*B*). The LO mode has a period of 25.7 fs ($\nu_{LO}$ = 38.9 THz), while the ZO mode has a slightly longer period of 41.1 fs ($\nu_{ZO}$ = 24.3 THz). Both of these modes are excited with amplitudes in the range of a few percent with respect to the lattice parameter and B–N bond length, which is experimentally feasible (42) and still leads to large and measurable effects. We also note that the phonon landscape is naturally anharmonic in hBN (43), which will slightly manifest in the results. The IR probe laser that drives HHG is assumed to have the following vector potential:

[Eq. 1] $$A(t)=A_{0}f_{\mathrm{env}}\left(t\right)\text{cos}\left(\omega t+\phi_{\mathrm{CEP}}\right)\hat{e}$$

where *A_0_* is the amplitude of the vector potential that is connected to the amplitude of the applied electric field, taken here as 0.27 V/Å (equivalent to 10^12^ W/cm^2^). This laser power is sufficient to induce extremely nonlinear HHG responses from hBN above the band gap but remains below the material damage threshold, as is required for time-resolved spectroscopy (44, 45). $\hat{e}$ in Eq. **1** is a linearly polarized unit vector, *f_env_(t)* is a dimensionless envelope function with a duration of ∼25 fs (see the *Materials and Methods* section for details), and ω is the fundamental carrier frequency corresponding to a wavelength of 1,600 nm. The temporal characteristics of A(t) are crucial because they outline a physical regime where the pulse duration is on the same order of magnitude as the phonon period. Moreover, a single period of the carrier wave has a duration of ∼5.3 fs, which is on the same order of magnitude as a quarter cycle of the phonon motion. Altogether, these conditions should lead to a strong modulation of the HHG response because within the IR pulse duration, the lattice undergoes less than a single cycle of ionic displacements, which breaks the time-translation symmetry that is exhibited by the phonon-free laser-matter system. On the other hand, the phonon motion is not considerably slower than changes in the laser envelope, which means that the dynamics cannot be described by a static approximation for the lattice. It is also noteworthy that for these parameters, the IR pulse is a multicycle pulse that usually does not lead to CEP sensitivity in the HHG spectra ($\phi_{\mathrm{CEP}}$ in Eq. **1** denotes the CEP that is taken as zero unless stated otherwise). We will show below that contrary to the standard case, such sensitivity is induced due to the presence of coherent phonon modes.

**Fig. 2.**
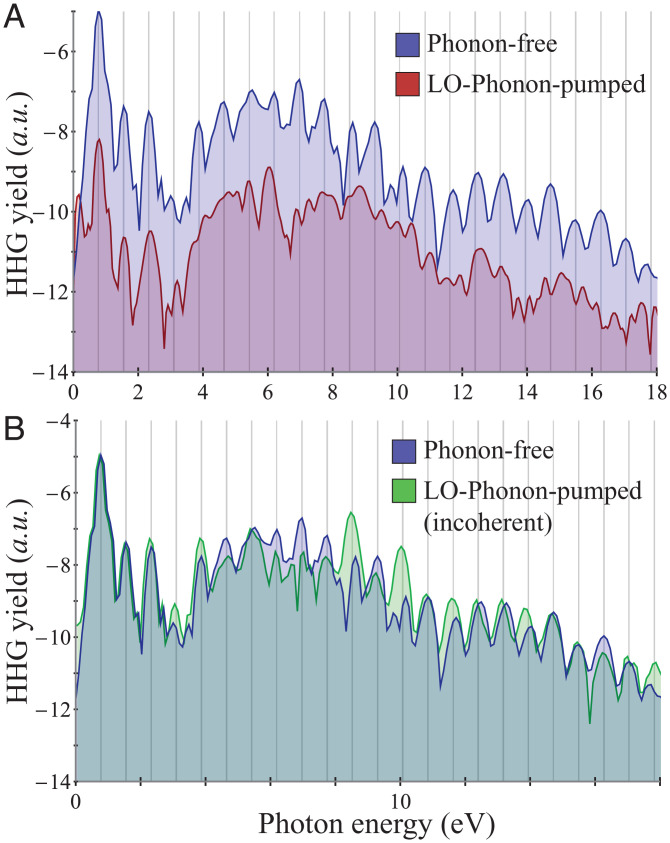
Effects of coherent phonons on HHG spectra. (*A*) HHG spectra with and without pre-excited LO phonon motion for IR laser polarized along the *x* axis (transverse to phonon motion). The HHG spectra in both cases are shifted from one another for clarity. (*B*) HHG spectra from incoherent LO phonon case compared to the phonon-free response. The incoherent case was calculated by averaging over a full cycle of the pump-probe delay. Gray lines indicate integer harmonic orders of the IR photon energy, and all spectra are presented in log scale. For the calculation, the LO mode was excited with an amplitude of 5.7% of the lattice parameter for bond stretching and 4.95% of the lattice parameter for bond compression.

Having outlined the setup, we explore HHG with a pumped LO phonon mode (similar results are expected from the degenerate transverse optical mode). Fig. 2*A* shows exemplary HHG spectra from the phonon-pumped (red) and phonon-free (blue) systems. Before addressing the dynamical response, it is worth analyzing the general structure of the spectra: in the absence of phonon dynamics, sharp harmonic peaks are obtained for both even and odd harmonics (because hBN is not inversion symmetric). The phonon-pumped system, on the other hand, shows a quasi-continuous plateau from 4 to 12 electron volts (eV) (note that small peaks in the spectra in this region do not appear at integer harmonic orders of the IR probe pulse), while perturbative emission up to the fourth order remains well defined. We also note a sharp peak resonant with the phonon frequency (at ∼0.15 eV), which is attributed to the LO phonon motion and the Born-Oppenheimer dynamics (i.e., the electron–phonon coupling induces a linear-response peak of the electronic current at the phonon frequency, where electrons slowly follow the lattice). The lack of frequency-comb harmonics in the higher-order response indicates that the temporal dynamics is no longer periodic due to the phonon-induced lattice displacement, as expected. The perturbative response, on the other hand, is more mildly modulated. Interestingly, if the HHG response is integrated over the pump-probe delay, the spectrum regains its frequency-comb nature with well-defined harmonic orders that are very similar to the phonon-free response (see Fig. 2*B*). This result is qualitatively equivalent to the thermal limit, where different regions in the lattice have random phonon phases that average out. It validates that in standard noncoherent conditions, it would be very difficult to determine experimentally the contributions of phonons to the response. It also shows that thermal phonons restore the time periodicity to the dynamics and have an isotropic nature (since sharp harmonic peaks are obtained), which makes them fundamentally different from the coherent phonons.

**Fig. 3.**
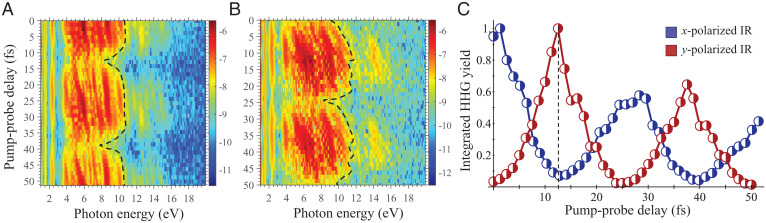
Temporally resolved HHG response with active LO phonons (*y*-axis motion). (*A*) HHG spectra versus pump-probe delay for *x*-polarized IR probe pulse. (*B*) Same as *A*, but for *y*-polarized IR probe pulse. The spectra are presented in log scale, and the HHG cutoff for the first plateau is indicated with dashed black lines. (*C*) Normalized HHG yield integrated over the plateau region versus the pump-probe delay for IR pulse polarized along the *x* axis and *y* axis. For the calculations, the LO mode was excited with an amplitude of 5.7% of the lattice parameter for bond stretching and 4.95% of the lattice parameter for bond compression. Dashed black line in *C* indicates the correspondence between the enhancement and suppression of the HHG yield, depending on the polarization axis of the IR pulse.

We next analyze the temporally resolved response in the LO phonon case. Fig. 3 *A* and *B* present the pump-probe-resolved HHG spectra for two probe laser polarizations (along the *x* axis, and *y* axis, respectively). As expected, the spectra are delay dependent, and there are strong modulations of the HHG yield that oscillate periodically with the phonon period. Most notably, there is sharp polarization sensitivity in this temporally resolved response: if the IR probe laser is polarized along the active phonon mode or transversely to it, the HHG yield is either enhanced or suppressed, respectively. We emphasize that this strong pump-probe delay sensitivity is obtained only if the probe pulse duration is on the same order of magnitude, or shorter, than the phonon period; otherwise, one effectively returns to the incoherent case (the HHG emission is effectively averaged over all pump-probe delays).

To further analyze these effects, we track a simple and more experimentally robust observable: the total integrated HHG yield in the plateau region. Fig. 3*C* presents the integrated HHG yield versus pump-probe delay, which oscillates periodically with a frequency $\nu_{LO}$. The yield modulates by factors of 5 to 10 depending on the delay. Further analysis shows that the minimal yield is obtained when the peak of the probe laser envelope roughly coincides with the maximally induced B–N bond stretching (that occurs at a pump-probe delay of ∼10.1 fs). The minimum is offset from the maximal bond stretching by ∼2.4 fs (it arises at a pump-probe delay of ∼12.5 fs). Similarly, the maximal yield is obtained when the peak of the laser envelope roughly coincides with the maximally induced B-–N bond compression. When the probe laser polarization axis is rotated to be transverse to the phonon motion (along the *x* axis), this dependence is exactly reversed (compare blue and red lines in Fig. 3*C*), which reflects the results in Fig. 3 *A* and *B*. This finding unambiguously establishes a real-space origin for the effect, because when the B–N bonds along the *y* axis are maximally compressed, those along the *x* axis are maximally stretched, and vice versa. Thus, the real-space displacement of the ions is directly imprinted on the temporally resolved HHG spectral response. A main result here is that the capability to temporally resolve the moment of maximal bond compression or stretching is limited by the duration and shape of the probe IR laser pulse, which, in this case, leads to the ∼2.4-fs error. We also note slight deviations from the perfect matching of peaks and minima in Fig. 3*C* at longer delays, which originate from higher-order effects of electron–phonon and phonon–phonon couplings as well as anharmonicity. These interesting phenomena are relatively small (<1 fs), such that they do not affect the main results derived here. Another prominent feature in Fig. 3*C* is a temporal damping of the HHG yield for longer delays. We believe that this effect arises from higher-order electron–phonon couplings, and it should be studied in future work.

We also highlight that there are some other effects induced in the HHG spectra by the coherent phonons that might prove useful for similar spectroscopic analysis, such as: 1) HHG cutoff modulations (see dashed black lines in Fig. 3 *A* and *B*), and 2) a linear energy shift in the harmonic emission energy with pump-probe delay (see plateau region in Fig. 3 *A* and *B*). The linear dependence of the HHG emission frequency on pump-probe delay likely results from phonon-driven phase factors in the electronic trajectories because the electrons effectively “feel” a temporally changing band structure due to the phonons, which depend on the pump-probe delay and can induce a phase shift in the trajectories.

**Fig. 4.**
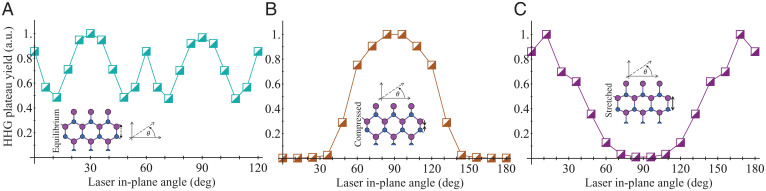
Polarization-resolved HHG response in ion-frozen hBN corresponding to the instantaneous lattice structure. (*A*) HHG plateau-integrated yield versus the laser polarization axis for the static lattice in the equilibrium geometry (due to the 3-fold rotational symmetry, only 120° are presented). (*B*) Same as *A*, but where the ions are statically frozen at a position with 4.95% bond compression for the B–N bond along the *y* axis. (*C*) Same as *B*, but where the ions are statically frozen at a position of 5.7% bond stretching for the B–N bond along the *y* axis. *B* and *C* correspond to the maximally displaced lattice structures along the LO phonon dynamics in Fig. 3*C*, where due to the reduced 2-fold rotational symmetry, only 180° are presented. (*Insets*) Schematic representations of the hBN structures (displacements of atoms in *B* and *C* are exaggerated for clarity). The axis system denotes the angle of the laser in-plane polarization (from the *x* axis in the geometries plotted). Each plot is normalized to maximal yield for clarity.

We now focus on the origin of the strong oscillations in the HHG yield with pump-probe delay. Fig. 4 presents the plateau-integrated HHG yield versus the laser polarization angle with respect to the *x* axis, which is calculated for three cases: 1) for the equilibrium configuration, 2) for a static lattice where the ions are displaced to maximally compress the B–N bonds along the *y* axis, and 3) for a static lattice where the ions are displaced to maximally stretch the B–N bonds along the *y* axis. In general, the HHG yield from hBN is highly anisotropic, and stronger emission is obtained when the laser is polarized along the B–N bonds even in the equilibrium system, as well as along other high-symmetry axes (Fig. 4*A*). This result is in line with previously observed anisotropies (46–49). When the lattice is distorted by a few percent (as occurs in the dynamical case), as in Fig. 4 *B* and *C*, this anisotropy is greatly enhanced, and the dominant HHG contributions arise when the laser polarization is parallel to the compressed B–N bond. This analysis verifies that the results in Fig. 3 that reflect the dynamical ion motion indeed probe the instantaneous changes in the lattice structure (because the dominant effect of HHG enhancement and supression in the temporal dynamics is reconstructed by just the static distorted lattice). It establishes an adiabatic picture for analyzing HHG yield modulations with respect to ultrafast structural distortions. We also note that an equivalent picture arises in *k* space, where the enhanced yield along the B–N bond can be rationalized due to instantaneous phonon-induced changes in the electronic band structure that couple to dominant interband HHG (2, 50) (see *SI Appendix*). Similar results are obtained for the ZO mode, though here, the dependence can be even stronger because ion displacement results in a large reduction of the band gap through the formation of midgap states (see *SI Appendix*).

At this point, it is worth mentioning that the strength of the effect (the depth of the HHG yield modulation with pump-probe delay) is connected to the amplitude of the excited phonon, where larger phonon amplitudes lead to more pronounced modulations (see *SI Appendix*). In particular, there is an exponential mapping between the HHG yield modulation and the pumped phonon amplitude, which physically originates from the instantaneous phonon-induced changes in the electronic band structure (e.g., a change in the band gap is exponentially mapped onto the tunneling probability from the valence to the conduction band, which is the first step in the HHG process). Moreover, the HHG cutoff (defined as the starting point of an exponential decay in the HHG yield) is modulated with the pump-probe delay (see Fig. 3 *A* and *B*). This clearly connects the emission to the instantaneous changes in the band structure and lattice geometry induced by the phonon motion, validating the adiabatic picture (see discussion in *SI Appendix*). The exponential connection between the HHG yield and the phonon amplitude could be used for the experimental reconstruction of the phonon amplitudes from HHG measurements as well (i.e., by performing several consecutive measurements with varying laser powers).

**Fig. 5.**
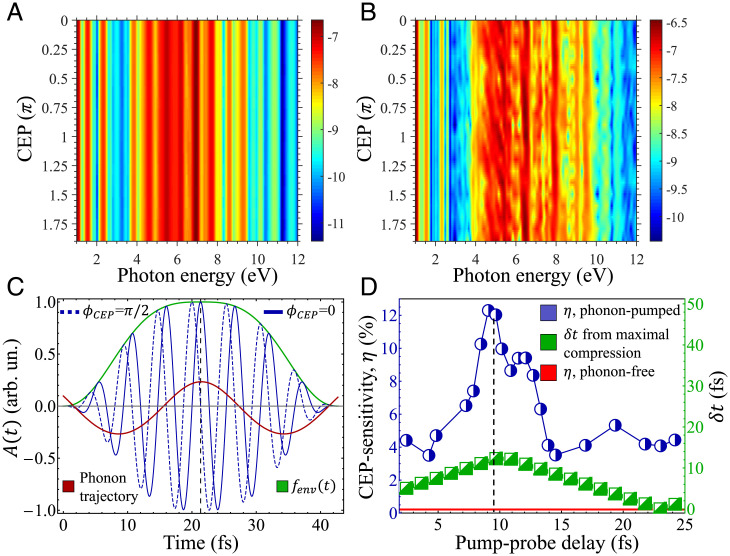
CEP-dependent HHG response for LO-driven coherent phonon dynamics. (*A*) CEP-dependent HHG spectra for the phonon-free static lattice in the equilibrium geometry (in log scale). No CEP dependence is present in the phonon-free case. (*B*) Same as *A*, but for the LO-pumped system for the pump-probe delay of 10.1 fs, showing onset of CEP sensitivity. (*C*) Illustration for the origin of the phonon-induced CEP sensitivity: the driving vector potential is illustrated for ϕ_CEP_ = 0 and ϕ_CEP_=π/2 in arbitrary units (in blue) and is presented in the same scale for the laser envelope function (green) and the phonon trajectory (red). A phase shift of π/2 in the CEP creates a temporal window of a quarter of a cycle that determines if the peak electric field is temporally aligned with the moment of maximal bond compression or if it is offset, leading to CEP sensitivity (black dashed line indicates this moment in time). (*D*) Degree of CEP sensitivity (η) in the phonon-pumped system versus pump-probe delay (blue line), on the same scale as the temporal distance between the moment of phonon-induced maximal B–N bond compression and the (*Right*) peak of the laser envelope, *δt* (green, corresponding to the *y* axis). The IR probe laser polarization is along the *x* axis, and the LO phonon is excited just as in Fig. 3. The red line indicates η for the phonon-free system as a reference. The dashed black line indicates the correspondence between the moment of maximal bond compression and the peak in η.

Up to this point, ϕ_CEP_ in Eq. **1** was assumed to be fixed to zero. We now explore the option of performing CEP-stable experiments in a multidimensional spectroscopy configuration—recording the HHG response versus the pump-probe delay versus the CEP. Fig. 5*A* presents the calculated HHG response versus CEP for the phonon-free system, while Fig. 5*B* presents the same spectra for one exemplary pump-probe delay in the LO phonon-pumped system. As seen in Fig. 5*A*, there is virtually no CEP dependence in the HHG spectra in the phonon-free system. This corresponds to the common understanding that CEP sensitivity should not arise from the multicycle pulses explored in our parameter regime. On the other hand, Fig. 5*B* shows that the HHG yield is modulated with the CEP when coherent phonons are present, especially in the plateau region. This establishes a mechanism by which phonons can be probed through HHG spectroscopy: the phonon mode introduces a longer timescale to the dynamics that temporally gates the laser electric field (see illustration in Fig. 5*C*). In that respect, the HHG process “senses” if this phonon gate is temporally synced with the peak or minima in the light wave because the corresponding electronic trajectories are driven in the instantaneous phonon-induced lattice structure. We emphasize that this CEP dependence is different in nature than those previously observed (e.g., in refs. 51–53) in the sense that the laser intensity is not modulated with the CEP (because the driving pulse has a long duration). This is why the HHG cutoff is largely insensitive to the CEP (unlike in the very short few-cycle pulse case), and the atto-chirp curves do not show their standard form. Similar nature results are obtained from other laser polarizations and from the ZO mode, indicating the generality of the result (see *SI Appendix*). Notably, thermal phonons are not expected to lead to such behavior because they do not have the correct symmetries to break the time periodicity of the dynamics (as illustrated in Fig. 2*B*). Thus, we expect that in this regime, thermal noise should be negligible for the measurement of phonon-induced CEP sensitivity. From similar considerations, we expect no CEP sensitivity if the probe pulse duration is much larger than the phonon period.

Interestingly, for our laser conditions, a CEP shift of π/2 creates a temporal window just 1.3 fs wide (a quarter cycle of the carrier wave period; see Fig. 5*C*), which could offer enhanced selectivity toward the instantaneous lattice distortion (this temporal resolution is disconnected from the total pulse duration and arises due to the CEP-sensitivity mechanism that is subcycle in nature). As an intuitive candidate for an observable that is capable of accessing this resolution (and can be readily measured in experiments), we consider the following observable: the normalized SD of the plateau-integrated HHG yield with respect to the CEP (denoted as η). η essentially quantifies the extent to which the HHG yield is CEP sensitive for a given pump-probe delay. For instance, for the phonon-free system, we find η≈0.25% (i.e., there is virtually no CEP sensitivity, in accordance with Fig. 5*A*). On the other hand, Fig. 5*D* presents η versus the pump-probe delay (blue line) for the LO-pumped system, which paints a different picture; there is relatively strong CEP dependence with η∼4% throughout, while for the particular delay of ∼9.5 fs, there is a large peak with η≈12% (this strong effect should be observable even in systems with few percent experimental uncertainty). We note that η should be periodic with the pump-probe delay for these conditions because the phonon trajectories here are only mildly affected by the electron–phonon couplings. Moreover, as it is a normalized quantity that estimates the degree of CEP sensitivity with respect to a baseline emission yield, it should be much less sensitive to background noises in the HHG yield and small modulations of the laser power.

Fig. 5*D* validates that η, indeed, provides enhanced sensing for the B–N bond compression: the peak at ∼9.5 fs occurs at the pump-probe delay for which the phonon-induced maximal bond compression essentially coincides with the peak of the pulse envelope (the temporal distance between the peak of the laser envelope and the phonon-induced maximal bond compression is denoted by δt and shown in green in Fig. 5*D*, whereas the phonon trajectories are proportional to a harmonic function of δt). In fact, the maximal bond compression is obtained at 10.1 fs, leading to an offset of just 0.6 fs (this can be compared to the peak in the HHG yield versus delay in Fig. 3*C*, which was offset by 2.4 fs, a factor of 4 larger). Remarkably, 0.6 fs is just ∼11% of a laser cycle duration, indicating the subcycle nature of the selectivity. The CEP-resolved spectra, thus, provide additional temporal information that was not present in the delay-dependent spectra, and overall exhibit enhanced selectivity and temporal resolution. Consequently, the multidimensional spectra can be used to “set the clock” to the exact moment of bond compression. This type of pump-probe-CEP multidimensional spectroscopy, therefore, offers enhanced sensing for ultrafast lattice distortions with resolutions of ∼1 fs. We also note in Fig. 5*D* the appearance of a minor secondary peak at a pump-probe delay of ∼12.2 fs that corresponds more closely to the one in Fig. 3*C*. The origin of this peak remains unclear, but we hypothesize that it corresponds either to slightly delayed electron dynamics driven in the system, or to a more subtle change in the electronic structure of the material during the bond stretching process. The exact nature of the effect should be explored in future work. Importantly, it highlights the ability of the multidimensional observable, η, to capture phenomena that are absent from the standard HHG response that are not CEP-resolved. Lastly, we highlight the extremely nonlinear nature of the multidimensional CEP-based spectroscopy technique: η in Fig. 5*D* does not show a peak at bond stretching that is equivalent to the one obtained upon bond compression. In that respect, the curve is highly nonlinear with particular sensitivity for the compression process. Consequently, the approach might be able to tap into the extreme nonlinearity of the HHG process for probing other processes as well (e.g., ultrafast phase transitions).

### Conclusions and Outlook.

To conclude, through ab initio calculations in hBN, we explored the possibility to probe phonons with pump-probe HHG measurements. We predicted several experimentally detectable effects that arise from coherently pumped phonons: 1) The HHG spectrum can form a quasi-continuous emission rather than discrete harmonic peaks due to the phonon timescale. We directly showed that this effect vanishes for incoherent phonons; 2) The harmonic yield in the plateau region is strongly delay dependent and oscillates periodically in accordance with the instantaneous structural changes in the lattice (such as bond compression and stretching); and 3) If the phonon motion has a timescale on the same order of magnitude as the pulse duration, CEP sensitivity emerges in the HHG spectra, which can be measured versus the pump-probe delay for an enhanced sensing of lattice distortions. Most importantly, the temporal resolution achievable via the CEP multidimensional spectroscopy is on the order of ∼1 fs and is well below the total pulse duration because it is inherently connected with subcycle dynamics (unlike standard pump-probe spectroscopy that is limited by the probe pulse duration and shape). Strikingly, we predict a temporal resolution of a quarter of a single laser cycle of the probe pulse.

The CEP multidimensional spectroscopy approach based on HHG has several attractive features. First, it allows obtaining subcycle temporal resolution without employing attosecond pulses or high-energy extreme ultraviolet photons as in other methodologies (54–58). Second, it allows a single method for simultaneously probing electron and lattice dynamics, which can be useful for several applications such as detecting electron–phonon couplings. Third, it opens possibilities for exploring the HHG mechanism itself by controlling the induced changes in the lattice, which should be useful for theory development in solid HHG.

While we limited our analysis to monolayer hBN (because it has relatively fast phonons that are easier to simulate and analyze), the discussed physical mechanisms are general and should apply to other materials as well, including bulk materials. In particular, it is possible that some of the CEP sensitivity observed in the paper by Schmid et al. (14) originated from a similar underlying mechanism (because in that work, a THz pump was used that could excite surface phonon motion). However, a more comprehensive study in Bi_2_Te_3_ would be required to determine such a possibility. Moreover, we emphasize that the phonon pumping method is versatile, and one could imagine also implementing this technique by pumping phonons with IR pulses as well as other excitation schemes (including phonons with finite momenta). All of these phenomena present exciting prospects for controlling the HHG emission as well as for the application of multidimensional HHG spectroscopy for probing phonons, lattice structural distortions, and phase transitions. Looking forward, we expect that our approach might also be applied for probing nonlinear phononics (22, 43), phonon–polariton motion in real time (59, 60), hyperbolic materials (61, 62), and real-time probing of electron–phonon coupling with possible applications for superconductivity.

## Materials and Methods

### Computational Details.

We report here on technical details for the calculations presented in the main text. We start by presenting our methodological approach that is based on TDDFT. All DFT calculations were performed using the real-space grid-based code, octopus (36–38). The KS equations were discretized on a Cartesian grid with the shape of the primitive lattice cell, where equilibrium atomic geometries and lattice parameters were taken at the experimental values. The *z* axis (transverse to the hBN monolayer) was described using nonperiodic boundaries with a length of 50 Bohr. Calculations were performed using the LDA for the exchange-correlation (XC) functional. Spin degrees of freedom and spin-orbit couplings were neglected. The frozen core approximation was used for core levels that were treated with appropriate norm-conserving pseudopotentials (41). The KS equations were solved to self-consistency with a tolerance <10^−7^ Hartree, and the grid spacing was converged to *Δx = Δy = Δz =* 0.3 Bohr. We employed a Γ-centered 36 × 36 × 1 *k*-grid, which converged the HHG spectrum and the phonon trajectories. We note that the relatively small grid spacing of 0.3 Bohr was required to obtain converged phonon trajectories.

For TDDFT calculations, we solved the time-dependent KS equations within the adiabatic approximation, represented in real space and in the velocity gauge, given in atomic units by

[Eq. 2] $$i\partial_{t}|\varphi_{n,k}^{KS}(t)>=\left(\frac{1}{2}{\left(-i\nabla +\frac{A\left(t\right)}{c}\right)}^{2}+v_{KS}\left(r,t\right)\right)|\varphi_{n,k}^{KS}(t)>$$

where $|\varphi_{n,k}^{KS}(t)>$ is the KS-Bloch state at *k*-point *k* and band *n*; A(t) is the vector potential of the laser electric field within the dipole approximation, such that $-\partial_{t}A\left(t\right)=cE(t)$; *c* is the speed of light in atomic units (*c* ∼ 137.036); and $v_{KS}\left(r,t\right)$ is the time-dependent KS potential given by

[Eq. 3] $$v_{KS}\left(r,t\right)=-\underset{I}{\sum }\frac{Z_{I}}{|R_{I}(t)-r|}\;+\;\int^{\text{​}}d^{3}r^{'}\frac{n\left(r^{'},t\right)}{|r-r^{'}|}\;+\;v_{XC}[n\left(r,t\right)]$$

where $Z_{I}$ is the charge of the *I*th nuclei and $R_{I}(t)$ is its coordinate, and $v_{XC}$ is the XC potential that is a function of $n\left(r,t\right)=\sum_{n,k}{|<r|\varphi_{n,k}^{KS}(t)>|}^{2}$, the time-dependent electron density. The KS wave functions were propagated with a time step of *Δt* = 0.2 a.u., which converged the HHG spectra. The initial state was taken to be the system’s ground state at the equilibrium geometry (i.e., $R_{I}(t=0)$ describes the lattice at equilibrium), which assumes that the Born-Oppenheimer approximation is valid prior to the turn on of the IR laser pulse. The propagator was represented by a Lanczos expansion. In the time-dependent calculations, we employed absorbing boundaries through complex absorbing potentials along the periodic *z* axis with a width of 12 Bohr, and ionization was well below 1% in all calculations.

The KS TDDFT equations of motion were coupled to classical phonons by the time-dependent ionic positions $R_{I}(t)$. The ion positions were allowed to evolve dynamically and were calculated by Ehrenfest dynamics as implemented in octopus code, where forces acting on the ions are evaluated directly from the electronic density and the laser (39). The initial condition for $R_{I}(t)$ was taken as the equilibrium geometry of the lattice, but where the ions were given initial velocities according to the particular phonon eigenmode (we verified that this approach is equivalent to starting the motion by displacing the ions directly and initiating the dynamics without an initial velocity). For the phonon-free calculations, $R_{I}$ was kept frozen, as is standardly done in TDDFT, either at the equilibrium geometry or at the distorted geometry as presented in the main text. We note that small errors in the ion trajectories were numerically observed, causing a drift of the hBN monolayer in space by up to 0.1 Bohr over 50 fs. We have verified that these errors arise due to the grid representation, which leads to small errors in the calculated ionic forces that build up over time for the long propagation durations. The contribution of this type of error to the HHG spectra was verified to be negligible since the total drift of the lattice in our conditions is small and slow. We note that in practical terms Eq. **3** has a slightly different form due to the utilization of pseudopotentials instead of performing all-electron calculations.

The time-dependent current expectation value was calculated directly from the time-dependent KS states as

[Eq. 4] $$J(t)=\frac{1}{\text{Ω}}\overset{}{\underset{\text{Ω}}{\int }}d^{3}r\;j(r,t)$$

where j(r,t) is the microscopic time-dependent current density

[Eq. 5], $$j(r,t)=\frac{1}{2}\underset{n,k}{\sum }\left[\varphi_{n,k}^{KS}{}^{*}(r,t)\left(-i\nabla +\frac{A\left(t\right)}{c}\right)\varphi_{n,k}^{KS}(r,t)+c.c.\right]$$

and Ω represents the volume integral over the primitive cell. An additional current term that arises from the pseudopotential nonlocal nature was also added to the current density in Eq. **5**. Note that the ionic current is neglected throughout this work because it is expected to dominantly contribute only at the phonon resonance frequency. The HHG spectrum was calculated as the Fourier transform of the first derivative of the current

[Eq. 6] $$I(\omega )=\;{\left|\int^{\text{​}}dt\partial_{t}\{f\left(t\right)\;J\left(t\right)\}e^{-i\omega t}\right|}^{2},$$

which was evaluated numerically with an eighth order finite-difference approximation for the temporal derivative and fast Fourier transforms. The HHG spectra in the paper only present the harmonic components within the hBN *xy* plane that propagate to the far field (i.e., any residual *z* currents that occur when there is ZO phonon motion are not plotted). Note that prior to calculating the HHG yield, the current was filtered with a temporal window similar to the laser field envelope (as in Eq. **6** above). This procedure greatly reduces any noise in the emission due to the phonon motion and suppresses the phonon-resonant peak.

The envelope function of the employed laser pulse *f_env_(t)* from Eq. **1** was taken to be of the following “supersine” form (63):

[Eq. 7] $$f_{\mathrm{env}}\left(t\right)={\left(\sin\left(\pi \frac{t}{T_{p}}\right)\right)}^{\left(\frac{\left|\pi \left(\frac{t}{T_{p}}-\frac{1}{2}\right)\right|}{\sigma }\right)}$$

where σ=0.75; and *T_p_* is the duration of the laser pulse, which was taken to be *T_p_ = 8T* (∼25 fs full-width half-max, where *T* is a single cycle of the fundamental carrier frequency that corresponds to 1,600 nm light). This form is roughly equivalent to a supergaussian pulse, but the field starts and ends exactly at zero amplitude, which is more convenient numerically.

## Supplementary Material

Supplementary File

## Data Availability

All study data are included in the article and/or *SI Appendix*.
