# Supplementary material for: Probing phonon dynamics with multidimensional high harmonic carrier-envelope-phase spectroscopy
Source: Proc Natl Acad Sci U S A. 2022 Jun 15;119(25):e2204219119. doi: 10.1073/pnas.2204219119 (PMC9231615; doi:10.1073/pnas.2204219119)
Supplement: Supplementary File [file pnas.2204219119.sapp.pdf]

PNAS

Supplementary Information for
Probing phonon dynamics with multi-dimensional high harmonic carrier envelope phase spectroscopy

Ofer Neufeld, Jin Zhang, Umberto De Giovannini, Hannes Hübener, and Angel Rubio

Corresponding authors: Ofer Neufeld and Angel Rubio.
Email: ofer.neufeld@gmail.com, angel.rubio@mpsd.mpg.de.

This PDF file includes:

Supplementary text
Figures S1 to S5

Supplementary Information Text

Additional results for zo phonon mode. We present here additional results complementary to those presented in the main text but for the ZO phonon-pumped system. Figure S1(a) presents the delay dependent HHG yield for ZO phonon amplitude of 11% out-of-plane motion with respect to the lattice parameter (note that the variation in the B-N bond lengths is much smaller due to the planar geometry of the lattice and amounts to ~1.8% in this case). Similar periodic dependence is observed, corroborating the results presented in the main text for the LO mode. Here the peaks and minima correspond to the lattice phonon-induced structural changes, where the ions are maximally stretched out-of-plane near the peak HHG yield, and are near the equilibrium position at the minimum emission. We also note that the HHG yield is modulated twice per phonon period due to the symmetry of this mode's motion – the HHG response is identical whether the N atoms move above the layer and the B atoms below it, or vice versa.

Figure S1(b) presents exemplary CEP-dependent spectra for the ZO driven case (in this case with a phonon amplitude of 19.3% of the lattice parameter for out-of-plane motion, corresponding to 5.4% maximal B-N bond stretching), showing that a similar CEP-sensitivity arises as was demonstrated in the main text. The emerging CEP sensitivity is even more pronounced for the ZO mode due to the larger changes it induces in the instantaneous band structure (as we show below). We have verified that similar in nature results are obtained also for other laser polarization and phonon amplitudes (not presented), validating the generality of the discussion in the main text.

Phonon-induced band structure changes. We present here an analysis of the phonon-induced changes to the electronic band-structure within a Born-Oppenheimer adiabatic picture. This complements the real-space analysis presented in the main text for the origin of the strong HHG selectivity to variations in the instantaneous B-N bond length.

Figure S2(a) presents the LDA-calculated band structure of hBN (KS eigenvalues) along selected high symmetry lines. Note that the plot includes the formally equivalent high symmetry points k={0.5,0} (the *M* point) and k={0,0.5} (denoted as *M''*), because these points become non-

equivalent in the distorted lattice that is no longer 3-fold symmetric (with a reduced instantaneous 2-fold symmetry for LO induced changes). In equilibrium, both the valence band maxima (VBM) and conduction band minima (CBM) are positioned at the *K* point, with a direct gap of 4.51 eV. Figures S2(b,c) present the calculated band structures on a similar level of theory, but where the ions have been displaced to their extrema position along the LO phonon trajectory (i.e. at maximal B-N bond compression and stretching, respectively, at the phonon amplitude of 5.7% (corresponding to the figures in the main text)). Several observations can be noted. Firstly, the band gap and positions of the VBM and CBM are different for bond stretching and compression – the CBM shifts to the *M* point for bond compression, or to the *M''* point for bond stretching. This is accompanied by a significant direct band gap closing at the *Γ* point, which is energetically very close to the VBM in both cases. Secondly, in both cases the direct optical gap is diminished compared to the equilibrium lattice, but it is more strongly reduced in the compressed structure (up to 4.34 eV).

The disparity between the band gap changes alone are not enough to explain the enhanced selectivity towards bond compression. This result could potentially be attributed to the induced changes in the directionality of the band structure. To further analyze the mechanism in *k*-space, we recall that the high harmonics in the energy region of ~10eV are above the band gap, and are likely generated by interband transitions with recombination surrounding the *Γ* point region (because those are the only regions allowing for high energy emission below the maximal band gap, see Fig. S2). This point of view assumes for simplicity that the main contribution to the plateau emission arises from the first valence and conduction bands. In the equilibrium lattice, this suggests that there should be stronger emission along lasers polarized from *K* to *Γ* (between the bonds, along the *x*-axis), because the initial excitation is preferred at the minimal band gap at the *K*-point, but for efficient HHG the conduction electrons need to be accelerated to regions near *Γ* (because those are the only regions with direct gaps of ~10eV in the plateau region). Similarly, emission for laser polarization from *M* to *Γ* (along the bonds, along the *y*-axis) should also be intense, because the direct gap at the *M* point is just a little bit higher than at *K*. This analysis fits well with the results in Fig. 4(a) in the main text, and thus we apply it also to the distorted lattices. For bond compression, the minimal optical gaps shifts to the M point, suggesting stronger emission along the *y*-axis. For bond stretching the minimal optical gap remains at the K, but is almost identical at the M'', both of which suggest stronger emission for lasers polarized along the *x*-axis. Of course, this simple picture is not quantitative and neglects other important features such as density of states and other regions in k-space that are not along high symmetry lines. Nevertheless, it roughly describes the *k*-space picture for the enhanced selectivity for just one of these processes depending on the laser polarization axis.

Figure S3 presents a similar analysis for the ZO mode. The main difference here compared to the LO mode is that this mode always stretches the BN bonds, and thus a contrast is imprinted onto the HHG response between the maximal stretching, and the equilibrium geometry. Notably, the ZO mode breaks other lattice symmetries than the LO mode, and allows for a mid-gap state to be generated that greatly reduces the band gap. As a result of this, there can be much larger HHG yield variations for the ZO-mode, and even enhanced CEP-sensitivity that is especially sensitive towards the formation of this mid-gap state at moment of maximal stretching. The HHG mechanism in the presence of the mid-gap has not been explored in this work, and will be topic of future studies.

HHG yield *vs.* phonon amplitude. We explore here the HHG yield modulation (the contrast obtained in Fig. 3(c) in the main text *vs.* pump probe delay) with respect to the pumped-phonon amplitude. Fig. S4 presents data of the integrated HHG yield in the first plateau region both from LO and ZO phonon modes. A clear exponential scaling is obtained in both cases (with R^2 values of 0.9954 and 0.9992, for the LO and ZO modes, respectively). This indicates that the mechanism behind the HHG yield modulation is connected to changes in the instantaneous electronic structure that is adiabatically attenuated with the lattice geometry (because changes in the band structure would map exponentially to the HHG yield due to the inherent extreme nonlinearity). The exact mechanism behind this effect is not yet known, but will be topic of future studies. We

hypothesize that it is connected to local changes in the electron density of sigma bonded electrons (in the B-N bonds) which might become more mobile under bond compression, or less under bond stretching.

Polarization sensitivity of perturbative harmonics. For completeness, we include here results similar to those in Fig. 4 in the main text, but for the polarization modulated harmonic power in the perturbative harmonics (harmonics 2, 3, and 4). Fig. S5 presents the integrated harmonic yield in the perturbative region (excluding the linear response peak). A similar trend to that in Fig. 4 in the main text is seen also in the perturbative regime. This is regardless of the fact that in monolayer hBN the perturbative harmonics are only weakly modulated with the laser polarization axis in the equilibrium geometry (at least in this examined regime). This indicates that the mechanism that modulates the HHG yield due to the phonon motion is general. Notably, the contrast in the yield modulation *vs.* laser orientation is slightly smaller for the perturbative harmonics compared to the higher harmonics, indicating that the extreme nonlinearity is playing an additional role.

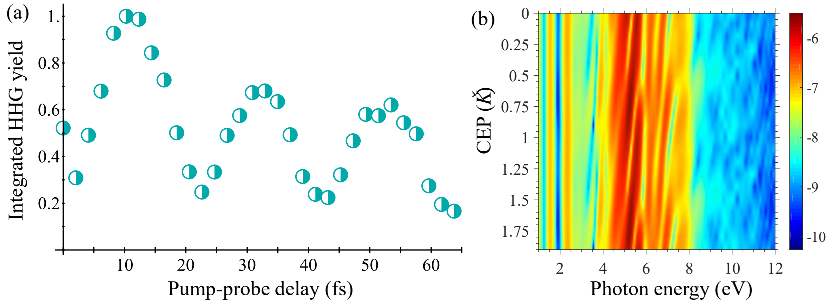

Fig. S1. HHG from ZO Phonon-pumped system. (a) Delay-dependent HHG plateau yield for out-of-pane phonon amplitude of 11% of the lattice parameter (1.8% bond stretching), and laser polarization along the *x*-axis. (b) Exemplary CEP-dependent HHG spectra for the ZO-pump system, in similar conditions to (a), but with a pump-probe delay of 9.3 femtoseconds and phonon amplitude of 19.3% for the out-of-plane motion (5.4% bond stretching). The spectra is presented in log scale.

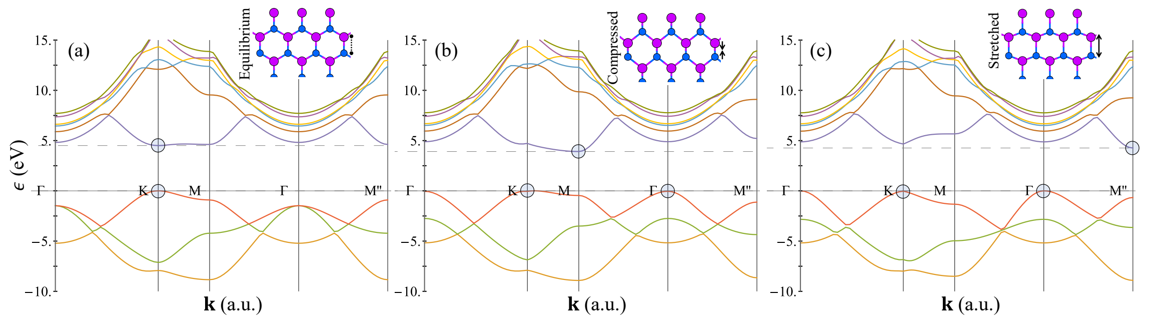

Fig. S2. LO Phonon-induced band-structure changes. (a) Equilibrium band-structure along high symmetry lines, calculated within LDA. (b) Same as (a), but where the ions are displaced by 5.7% towards compression of the y-axis B-N bonds. (c) Same as (b) but for bond stretching of 4.95%. The highest occupied and lowest unoccupied levels are denoted with horizontal dashed lines, the VBM and CBM are denoted with blue circles, and insets represent the lattice geometry.

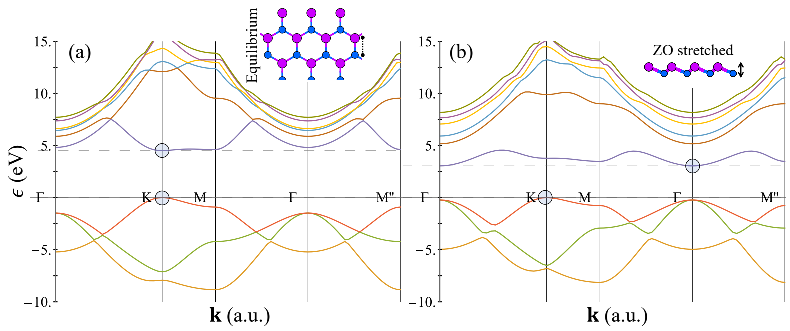

Fig. S3. ZO Phonon-induced band-structure changes. (a) Equilibrium band-structure along high symmetry lines, calculated within LDA. (b) Same as (a), but where the ions are displaced by 5.4% towards the maximal out-of-plane bond stretching. The highest occupied and lowest unoccupied levels are denoted with horizontal dashed lines, the VBM and CBM are denoted with blue circles, and insets represent the lattice geometry.

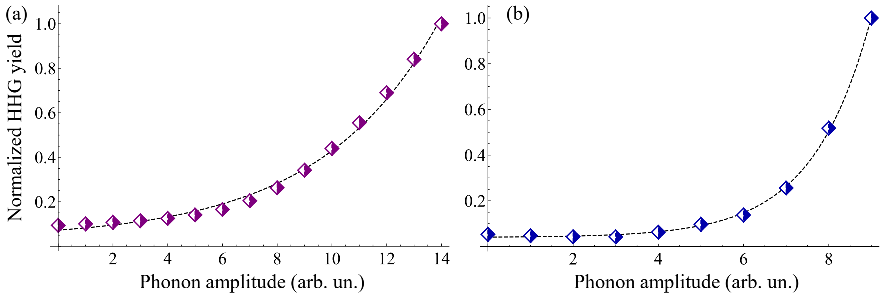

Fig. S4. HHG yield modulation *vs.* phonon amplitude. (a) Calculations for LO mode in hBN in the same conditions as Fig. 2(a) in the main text, but where the phonon amplitude is increased. (b) Same as (a) but for the ZO mode with conditions similar to those in Fig. S1. Dashed lines indicate exponential function best fits.

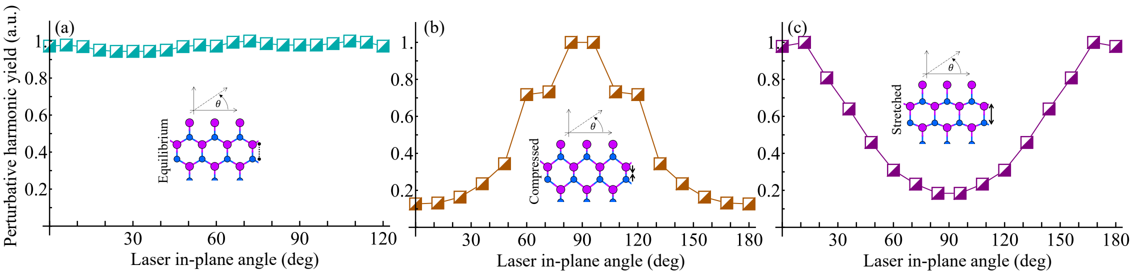

Fig. S5. Perturbative harmonic yield modulation *vs.* laser in-plane orientation in the static case for: (a) the equilibrium lattice, (b) the compressed lattice, and (c) the stretched lattice. The lattice geometries and laser conditions correspond to those in Fig. 4 in the main text. The insets correspond to the insets in Fig. 4 in the main text.
